# The Role of the ACE2/MasR Axis in Ischemic Stroke: New Insights for Therapy

**DOI:** 10.3390/biomedicines9111667

**Published:** 2021-11-11

**Authors:** Mansoureh Barzegar, Karen Y. Stokes, Oleg Chernyshev, Roger E. Kelley, Jonathan S. Alexander

**Affiliations:** 1 Molecular and Cellular Physiology, Ochsner-LSU Health Sciences Center, Shreveport, LA 71130-3932, USA; Mansoureh.barzegar@lsuhs.edu (M.B.); karen.stokes@lsuhs.edu (K.Y.S.); 2 Neurology, Ochsner-LSU Health Sciences Center, Shreveport, LA 71130-3932, USA; oleg.chernyshev@lsuhs.edu (O.C.); roger.kelley@lsuhs.edu (R.E.K.); 3 Medicine, LSU Health Center, 1501 Kings Highway, Shreveport, LA 71130-3932, USA; 4 Oral and Maxillofacial Surgery, Ochsner-LSU Health Sciences Center, Shreveport, LA 71130-3932, USA

**Keywords:** ischemic stroke, angiotensin converting enzyme-2, MasR, therapy

## Abstract

Ischemic stroke remains the leading cause of neurologically based morbidity and mortality. Current stroke treatment is limited to two classes of FDA-approved drugs: thrombolytic agents (tissue plasminogen activator (tPA)) and antithrombotic agents (aspirin and heparin), which have a narrow time-window (<4.5 h) for administration after onset of stroke symptoms. While thrombolytic agents restore perfusion, they carry serious risks for hemorrhage, and do not influence damage responses during reperfusion. Consequently, stroke therapies that can suppress deleterious effects of ischemic injury are desperately needed. Angiotensin converting enzyme-2 (ACE2) has been recently suggested to beneficially influence experimental stroke outcomes by converting the vasoconstrictor Ang II into the vasodilator Ang 1–7. In this review, we extensively discuss the protective functions of ACE2-Ang (1–7)-MasR axis of renin angiotensin system (RAS) in ischemic stroke.

## 1. Introduction

Historically, ‘stroke’ is defined as the sudden or rapid onset of neurological deficits caused by acute vascular hypoperfusion and distress with the central nervous system (CNS), which lasts longer than 24 h [1,2]. Most stroke patients exhibit significant motor weakness and sensory disturbances, reflecting non-function and/or destruction of millions of neurons [3]. Risks for stroke can be classified as ‘modifiable’ (e.g., hypertension, diabetes, smoking), or fixed (such as atrial fibrillation and transient ischemic attacks (TIA)) [3]. Modifiable risk factors are more common and provide significant opportunities to reduce population risks through lifestyle changes. Fixed risk factors are less prevalent and are usually harder to manage [3]. The primary vascular disturbances which provoke stroke are thrombotic (cerebral infarction) and hemorrhagic (intracerebral (ICH) and subarachnoid hemorrhages (SAH)) [2,4]. Despite its global effect, the term “*stroke*” is not uniformly clear in clinical research. The term “stroke” was initially coined in medical terminology in 1689 by Dr. William Cole, in *A Physico-Medical Essay Concerning the Late Frequencies of Apoplexies*. Prior to Cole, the common term used to describe acute non-traumatic brain injuries was “*apoplexy*”, which had been in use since Hippocrates of Kos, ~400 BC [2]. Following introduction of “stroke”, clinicians have tried to improve upon the specificity of the expression, and in the mid-20th century, a newer and more distinct term for non-permanent vascular-related episodes of brain dysfunction was introduced: “*transient ischemic attack*” (TIA). In 2009, based on advances in basic science, neuropathology, and neuroimaging, an expert committee of the American Heart Association (AHA)/American Stroke Association (ASA), updated the definition of TIA as a “transient episode of neurological dysfunction caused by focal brain, spinal cord, or retinal ischemia without acute infarction” [2]. A year later, the International Classification of Diseases (ICD) system distinguished cerebrovascular stroke disorders as TIA, cerebral ischemic stroke, intracerebral hemorrhage (ICH), and subarachnoid hemorrhage (SAH) [2].

Despite many advances in management, ischemic stroke still has few therapeutic treatments. The only FDA approved drug for ischemic stroke tissue plasminogen activator tPA, needs to be administered within 4–5 h after onset of stroke symptoms to be effective. Antiplatelets (aspirin) and anticoagulants (warfarin) prevent the secondary stroke but carry risks for intracerebral hemorrhage. While thrombolytic and antithrombotic agents restore perfusion, the ensuing and progressive vasoconstriction that follows stroke provokes irreversible brain microvascular injury, which greatly intensifies stroke severity and worsens outcomes. The lack of effective treatments has motivated researchers to seek alternative therapeutic approaches, which reduce cerebral stress before and beyond this window. In this review, we discuss the different types of strokes, pathology of stroke, current treatment strategies, and their limitations. Furthermore, we introduce a recently appreciated role for the angiotensin converting enzyme-2 (ACE2)/Ang (1–7)/MasR axis of renin angiotensin system (RAS) in stroke, and its therapeutic potential in stroke therapy.

## 2. Classification of Stroke Subtypes

**Figure 1:**
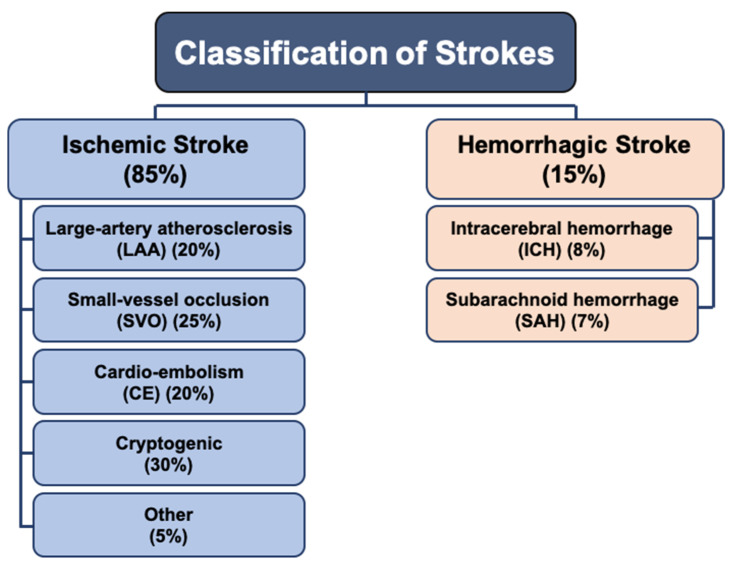
Classification of stroke subtypes. Stroke is classified into two major forms—ischemic (85%) and hemorrhagic (15%) groups.

The ‘Trial of Org 10172 in Acute Stroke Treatment’ (TOAST) criteria, the most frequently used and agreed upon system, identified the most likely pathophysiological mechanisms based on clinical findings and results of investigations, and classified strokes into two major ischemic and hemorrhagic groups (Figure 1) [3]. In general, ischemic strokes represent 80%–85%, while hemorrhagic stroke accounts for the other 15%–20%, of all strokes [5,6,7].

### 2.1. Ischemic Stroke and Transient Ischemic Attack

Ischemic stroke is caused by a sudden obstruction of one or more cerebral arteries due to either thrombosis or embolus, and provokes an acute loss of neurons, astroglia, oligodendroglia, and disruption of cortical synaptic structure [2,8]. The associated symptomatic cerebral ischemic events last longer than 24 h, whereas such strokes are considered to be transient ischemic attacks (TIAs) when these events last 24 h or less [3]. Following transient or permanent loss of blood flow, the center of the ischemic zone, where there is severe hypoperfusion and neurons die, is known as the “*infarct core*”. The ischemic core contains unsalvageable dead tissue and represents the terminal events of the ischemic cascade [3]. The core area is surrounded by the “*ischemic penumbra*”, which is also hypoperfused tissue but is an area where cells are still metabolically active and may be ‘salvageable’ [9,10]. In the ischemic penumbral region, neuronal damage develops more slowly because collateral flow from adjacent vascular areas can provide cerebral perfusion above the threshold required for cell survival [11]. Consequently, rescuing the ischemic penumbra is considered a major therapeutic target for stroke intervention because it holds the greatest promise for restoring neurological outcomes [3].

According to the Trial of Org 10172 in Acute Stroke Treatment (TOAST) classification system, ischemic stroke can be sub-divided into five categories [12,13]:

Large-artery atherosclerosis (atherothrombosis) (LAA): LAAs mostly result from atherosclerotic and thrombotic products (so-called artery-to-artery embolism). LAA is classified into two subtypes: extracranial and intracranial. Extracranial LAA is caused by stenosis or infarction distal to major brain arteries, and includes aortic arch, ascending aorta, and the extracranial carotid artery. In the intracranial LAA, ischemia occurs in the arteries proximal to the Circle of Willis13.

Cardioembolism (CE): this category includes ischemic strokes with arterial occlusions due to an embolus originating from the heart. The most known etiologies of cardioembolic stroke include atrial fibrillation, valvular heart disease, large myocardial infarction (MI), and dilated cardiomyopathy [13].

Small-vessel occlusion (SVO): stroke attributed to SVO, which is also referred to as ‘lacunar’ stroke, is defined by anatomical location. Typical underlying pathologies include microatheroma of the penetrating arteries, lipohyalinosis, microembolism, and branch occlusive disease [13].

Stroke of undetermined cause (cryptogenic stroke): strokes are designated in this category if none of the above causes of cerebral infarction could be determined [5], mostly likely due to lack of available diagnostic tools, or the restrictive definition of atherothrombotic causes.

Strokes of “other” determined cause: this term refers to nonatherosclerotic vasculopathies, hypercoagulable states, and hematologic disorders, as well as stroke in the setting of migraine [5].

### 2.2. Hemorrhagic Strokes

Hemorrhages in the CNS are considered as strokes if they are non-traumatic and caused by a vascular injury to the CNS [2]. There are two types of hemorrhagic stroke: intracerebral hemorrhage and subarachnoid hemorrhage.

Intracerebral hemorrhage (ICH): in ICH, there is rapid development of neurological dysfunction due to the focal collection of blood within the parenchyma or ventricular system of brain. Non-traumatic ICH is mostly associated with underlying vascular abnormalities such as preexisting hypertension and anti-coagulation therapy [14].

Subarachnoid hemorrhage (SAH): non-traumatic SAH is defined by bleeding in the subarachnoid spaces due to vascular causes e.g., cerebral aneurysm rupture, arteriovenous malformation, intracranial artery dissections, mycotic aneurysms, and bleeding disorders [15].

## 3. Current Treatment and Prevention Strategies

In general, one of the primary goals in stroke prevention is to manage the major vascular risk factors, including hypertension hyperlipidemia, diabetes mellitus, obesity, diet and nutrition, and tobacco use [13,16]. However, once an individual has a stroke, perhaps the most remarkable advances in the management of acute stroke have been the routine strategies used in stroke care units, the most effective practices being improved blood pressure control, early mobilization, and identification of the stroke etiology [3]. As can be appreciated from the information below, knowing the stroke etiology is a critical step in the treatment of stroke, as well as prevention and reduction of future strokes. Thrombolytic treatment is the only FDA approved approach for acute ischemic stroke. Intravenous administration of biologically effective thrombolytic agents, e.g., tissue plasminogen activator (tPA), within 3–4 h after symptom onset, reduces neurological deficits and improves functional outcomes of stroke patients [3,17]. However, this improvement is mostly achieved at the expense of intracranial hemorrhages [18], which are seen in approximately 6–7% of cases, but are increased by older age, high blood pressure, very severe neurological deficits, and severe hyperglycemia [3,17]. Moreover, due to the short therapeutic time window, the number of patients who might potentially benefit from this treatment is small [3]. Antiplatelet therapy with aspirin is mostly used in patients without a cardioembolic cause of stroke, and has proven to be an appropriate approach for secondary stroke prevention in the case of intracranial large artery atherosclerosis (LAA) and small artery occlusion (SAO) [13,16]. The advantages of using aspirin include low cost, ease of oral administration, and lower toxic side effects [3]. Clopidogrel, another antiplatelet drug, significantly improves ischemic stroke outcomes, although it is not effective in reducing recurrent strokes [16]. Clopidogrel monotherapy is slightly more effective than aspirin against vascular ischemia affecting the heart and the peripheral circulation [3]. In cases where cardioembolism is responsible for strokes, anticoagulant therapy with Warfarin is the most endorsed strategy in secondary prevention, especially in patients with atrial fibrillation [3,13]. However, there is evidence that Warfarin use can be accompanied by side effects such as major bleeding (intracerebral hemorrhage) [3]. In addition to pharmacological approaches, a number of surgical interventions can prove beneficial. In particular, thrombectomy devices have been developed to physically remove the blood clot/embolus from major vessels, including middle cerebral and basilar arteries [3]. Primary occlusion in the brain can lead to cerebral edema and elevated intracranial pressure, both of which result in secondary brain damage and poor outcomes. Decompressive craniectomy is another surgical method, in which the skull is opened to relieve intracranial hypertension [19]. The effectiveness of decompressive surgery has been most evident in young patients with malignant middle cerebral artery infarction and life-threatening brain edema [3].

Although there have been advances in management, stroke still has many therapeutic challenges. One of the FDA approved drugs, tPA, has a very narrow therapeutic window (<4 h) for administration after onset of stroke symptoms to be effective. Conversely, antithrombotic agents prevent the recurrence of stroke, while carrying serious risks for hemorrhage. Neither of these interventions target events in reperfusion injury, i.e., loss of blood-brain barrier (BBB) and immune cell infiltration, which worsen stroke outcomes. Therefore, stroke therapies that target such deleterious acute effects of ischemia-reperfusion injury are desperately needed.

Recently, stem cell-based therapies (SCT) have been intensively studied as a powerful and promising set of approaches, which may improve ischemic stroke outcomes. In our previous study, we showed that cerebral blood flow (CBF), infarct size, and neurological outcomes in our ischemic stroke model were dramatically and significantly improved by human placenta mesenchymal stem cells (hPMSCs) in a stem cell ACE2-dependent/vascular MasR dependent fashion [20].

## 4. ACE2/Angiotensin (1–7)/MAS Receptor Axis in Ischemic Stroke

Our understanding of the renin angiotensin system (RAS) conventionally holds that angiotensin I (Ang I) is converted into angiotensin II (Ang II) by the enzymatic action of angiotensin converting enzyme-1 (ACE) [21]. Under normal physiological conditions, Ang II then activates angiotensin type 1 receptor (AT1R) in several tissues to increase blood pressure and stimulate electrolyte and water retention by the kidney. However, prolonged Ang II signaling and overstimulation of AT1R provokes inflammation, fibrosis, and cellular hypertrophy, all of which contribute to stroke pathophysiology [21,22].

In contrast to the deleterious actions of ACE-Ang II-AT1R, the angiotensin converting enzyme-2/angiotensin 1–7/Mas receptor (ACE2/Ang (1–7)/MasR) axis, a recently discovered arm of the renin angiotensin system (RAS), has been shown to beneficially influence outcomes in experimental stroke models and other cardiovascular diseases [23,24,25]. The ACE2/Ang (1–7)/MasR pathway has many characteristics which make it an attractive potential target to induce neuroprotection in the context of stroke [21] and, importantly, all of the components of the ACE2/Ang (1–7)/MasR axis are already expressed by cells within the CNS [21,22]. Endogenous ACE2 has been found in the cerebral cortex, while MasR is present on neurons, microglia, and some endothelial cells in healthy brains [23].

**Figure 2:**
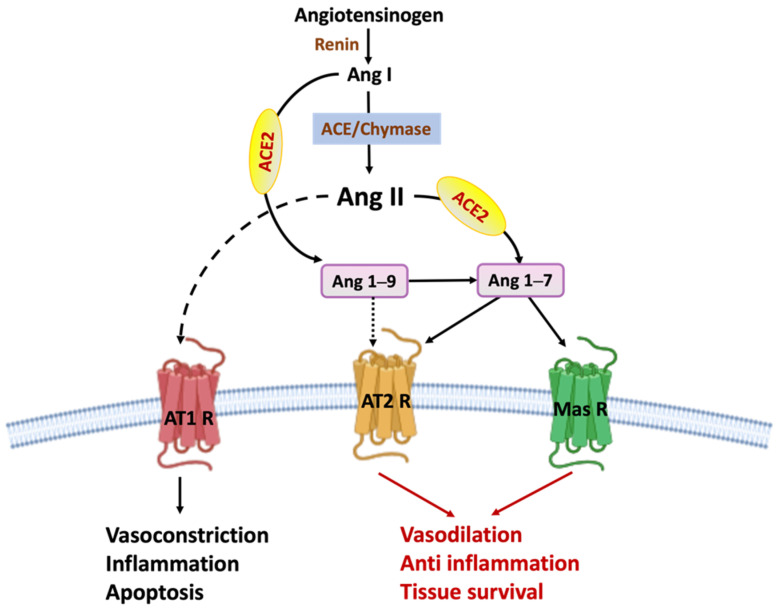
Classical and alternative pathways of the renin angiotensin system (RAS). In the classical axis of RAS, angiotensin-converting enzyme (ACE) converts angiotensin I (Ang I) into angiotensin II (Ang II). Ang II activates angiotensin type 1 receptor (AT1R) to trigger vasoconstriction, inflammation, fibrosis, and cellular hypertrophy, and apoptosis. In the alternative axis, ACE2 converts Ang II into Ang (1–7) or Ang I into Ang (1–9). Ang (1–9) is converted to Ang (1–7) by the enzymatic activity of ACE or neprilysin. Ang (1–7) binds to either AT2R or MasR to exert vasodilator, anti-inflammatory, and anti-apoptotic effects.

The ACE2 enzyme is associated with the endothelial surface, and ACE2 activity shifts the ACE/Ang II/AT1R axis to ACE2/Ang (1–7)/MasR by converting the vasoconstrictor Ang II to Ang (1–7) [26]. In addition, ACE2 converts Angiotensin I to Ang (1–9), which in turn also yields Ang (1–7). Ang (1–7) exerts potent vasodilatory, anti-inflammatory, and anti-proliferative effects by binding to MasR and the angiotensin type 2 receptor (AT2R) [26,27,28]; thus, this ACE2-induced shift in the ‘balance of power’ from Ang II to Ang (1–7) prevents post-stroke vasoconstriction and the pro-thrombotic and vascular inflammatory phenotype (Figure 2).

### 4.1. ACE2/Ang (1–7)/Mas R Axis Components in the Brain

ACE2 gene and protein. ACE2, discovered and named in 2000, shares ~40% identity and ~61% similarity in amino acid sequence with ACE [29]. ACE2 is a 120 kD metalloprotease consisting of a single zinc-binding domain [29] which is expressed as a transmembrane protein. There is also a soluble and truncated form of ACE2, lacking the transmembrane and cytosolic domains but retaining catalytic activity. Originally, ACE2 protein was predominantly described in the endothelium of various vessels in the heart and kidney [29]. However, low levels of ACE2 mRNA have also been detected in vascular smooth muscle, glia, neurons, and endothelial cells of the human brain [22].

The primary function of ACE2 is to directly convert Ang II into the vasodilatory heptapeptide Ang (1–7) and phenylalanine as it has a 400-fold higher affinity for Ang II than for Ang I [22,29]. Additionally, ACE2 as a carboxypeptidase cleaves the COOH-terminal leucyl residue from decapeptide Ang I to produce Ang (1–9), which has been recently shown to inhibit the action of ACE [30]. Ang (1–9) can then be converted to Ang (1–7) by ACE or neprilysin (also known as neutral endopeptidase) [29,31]. One in vitro study has reported that ACE2 can also cleave des-Arg-bradykinin, apelin fragments, and neurotensin [29].

Ang (1–7) production and metabolism. Ang (1–7) has been detected in the hypothalamus, medulla oblongata, amygdala, and blood vessels in the brain [29]. In addition to the pathways already described, Ang (1–7) can be formed directly from Ang I by prolyl-endopeptidase or neprilysin [24,29]. It is reasonable that Ang (1–7) synthesis takes place primarily in the extracellular space since the catalytic site of ACE2 is located outside the cell, however, it may occur in extracellular fluid because ACE2 can be released from the cell surface by ADAM-17. Endocytosis of truncated form of ACE2 might lead to formation of intracellular Ang (1–7) [29]. Additionally, endocytosis of ACE2 is known to be induced by AT1R activation by Ang II suggesting that there is crosstalk between Ang II and Ang (1–7)/Ang (1–9) that leads to antagonism of the opposite pathway. Ang (1–7) is hydrolyzed by amino-peptidases generates Ang (2–7) and Ang (3–7) [30]. Ang (1–7) can also be metabolized to Ang (1–5) by ACE, or to Ang (1–4) by neprilysin [29]. While manipulation of Ang (1–7) has enormous therapeutic potential for stroke and other diseases, it is rapidly degraded in the stomach when administered orally; therefore, Ang (1–7) is considered orally inactive. Special formulations to overcome this limitation, other routes of administration, or different approaches to upregulate the ACE2/Ang (1–7)/MasR axis may be required in order to harness its beneficial effects as a treatment for stroke.

Ang (1–7) receptors. Two important receptors might be activated by the ACE2 pathway and contribute to stroke/MCAO protection: the AT2 and Mas receptors. On the vascular side, blood vessels are major sites for formation and biological actions of Ang (1–7) [29]. Ang (1–7) stimulates prostaglandin and NO release from endothelial cells, enhances bradykinin actions (via inhibition of ACE activity), and inhibits vascular smooth muscle cell (VSMC) growth [29], actions which are considered to be stabilizing and homeostatic. The Ang (1–7) heptapeptide appears to exert its protective function mainly by binding to and activating a unique G-protein coupled receptor known as the Mas receptor [21,24]. Importantly, the ACE2-Ang (1–7)-MasR axis of RAS is considered to be a very significant antagonist to Ang II and counteracts numerous physiological and pathophysiological effects of the ACE-Ang II-AT1R axis [23,24].

Angiotensin receptor 2 (AT2R). Tissue levels of AT2R are increased in the peri-infarct region in the brain following ischemic insult, and recent studies reported that activation of AT2R may even exert beneficial effects against ischemic stroke [22]. Within the brain, there is evidence that AT2R activation may result in differentiation and regeneration of neurons, which is critical for the neurotrophic and protective actions mediated by AT2R [22]. On the vascular side, AT2R mediates vasodilatory pathways through the production of bradykinin, NO, and cGMP [32], thus helping restore and maintain blood flow after stroke.

Mas receptor (MasR). The MasR was originally identified in 2003 as an orphan proto-oncogene, reflecting its tumorigenicity in nude mice [22,29]. The mRNA expression of MasR has been detected predominantly in hippocampus, amygdala, cortex, olfactory bulbs, and thalamus areas of the brain and to some extent in kidney and heart [22,33]. Using immunostaining, dense expression of MasR protein has been observed in vascular-related areas from the medulla to the forebrain, such as the nucleus of the tractus solitarius (NTS), rostral ventrolateral medulla (RVLM), caudal ventrolateral medulla (CVLM), supraoptic nucleus, and lateral preoptic area (previously shown as the target site for the action of Ang (1–7) in the brain) [29]. At the cellular level, immunohistochemical analysis has shown the presence of MasR on neurons, microglia and endothelial cells of the cortex, basal ganglia, thalamus, hypothalamus, hippocampus, and striatum [22]. MasR protein consists of seven hydrophobic transmembrane domains, and hydrophilic NH2- and COOH-terminal ends. This receptor shares similarity with the G-protein-coupled receptor subfamily [29]. Ang (1–7) is the primary ligand that binds to MasR.

### 4.2. ACE2/Ang (1–7)/MasR Downstream Signaling

The classical Ang II-mediated RAS downstream signaling pathway is initiated by binding of Ang II to the AT1 receptor leading to the activation of G_q_-mediated phosphoinositide hydrolysis, which results in increased intracellular Ca^2+^ followed by activation of protein kinase C (PKC) and Ca^2+^/calmodulin-dependent protein kinase II (CaMKII) [29]. These signaling kinases inhibit K^+^ currents and activate Ca^2+^ currents, consequently inducing neuronal depolarization and sympathetic outflow. Moreover, PKC can stimulate the formation of reactive oxygen species (ROS) by activating NADPH oxidase [29]. Ang II can also act through phospholipase C to activate the Ras/Raf/MAPK pathway. This results in the phosphorylation of transcription factors, e.g., c-Jun and c-Fos to promote the transcription of genes involved in the synthesis and transport of norepinephrine (NE) in neurons and thus, sympathetic outflow.

It is now understood that Ang (1–7) opposes Ang II-mediated signaling cascades through activation of an independent set of signaling molecules [29,30]. The first evidence from the brain indicated that NO is the major downstream effector upon Ang (1–7) receptor activation, as Ang (1–7) colocalized with nitric oxide (NO) synthase in neurons and endothelial cells [29]. NO, an ubiquitous gasotransmitter, is a crucial mediator which improves neuronal survival, maintains endothelial function and vasodilator tone, inhibits platelet aggregation and neutrophil adhesion, and modulates VSMC contraction and proliferation [34,35]. Several groups have shown that AT2R and bradykinin B2 receptors are also involved in NO release via activation of cGMP/PKG signaling pathway [29,36]. NO mediated increases in cytosolic cGMP concentration lead to PKG activation which could phosphorylate both voltage dependent Ca^2+^ channel and synaptic vesicle proteins associated with neurotransmitter release [36]. It should be noted that depending on the NOS isoform involved in NO production, NO may be neuroprotective or neurotoxic; as small fluxes of NO produced by endothelial NOS (eNOS) inhibit apoptosis, whereas large NO fluxes from inducible NOS (iNOS) may induce apoptosis [35].

It has also been reported that Ang (1–7)-mediated activation of the PI3 kinase/Akt/PKB pathway results in long-lasting phosphorylation (on Ser^1177^ residue) and activation of eNOS following focal cerebral ischemia-reperfusion in rat models [29,34,35], providing evidence that Ang (1–7) also enhances NO production through a Ca^2+^-independent pathway. Although eNOS regulation via the bradykinin B_2_ receptor also partially depends on the PI3K/Akt pathway, a greater portion appears to be mediated through a calmodulin K-II and Ca^2+^ dependent mechanism [34]. The relationship between Ang (1–7) and bradykinin is even more complex than these receptors signaling through the same pathways: as illustrated in cerebral ischemia, Ang (1–7) administration significantly enhanced mRNA and protein expression of bradykinin and the B2 receptor [29,37].

Overexpression of ACE2 in both endothelial cells and neurons resulted in enhanced eNOS phosphorylation of Ser^1177^, and reduced phosphorylation of Thr^495^ (the site for negative regulation of eNOS), thereby promoting NO release [29,34]. An increased ratio of AT2R/AT1R and MasR/AT1R in the brain may also be involved in the formation of NO [23,29]. Additionally, ACE2 has been shown to reduce oxidative stress in human endothelial cells either by preventing Ang II-mediated upregulation of p22^phox^ (major subunit of NADPH oxidase) or by promoting Ang (1–7)-mediated activation of downstream antioxidant signaling pathways [29]. Ang (1–7) also influences other signaling molecules and peptides including arachidonic acid, prostaglandins, and endothelium-derived hyperpolarizing factor (EDHF) [29,30]. Prostaglandin release causes vasodilation, inhibition of cell growth, and opposes AT1R-mediated harmful effects of Ang II [34].

### 4.3. ACE2/Ang (1–7)/Mas R Axis and Ischemic Stroke Neuroprotection

Direct evidence for a protective role of ACE2 in stroke includes prevention of cerebral damage and behavioral dysfunction in the endothelin-1 (ET-1)-induced MCAO model by administration of an ACE2 activator Diminazene aceturate. Ang 1–7 appears to exert several neuroprotective functions in the context of stroke (*discussed below*). It has been shown that the level of Ang 1–7 in both serum and the brain cortex is increased during the early hours following MCAO in rats, and this is accompanied by increased expression of ACE2 and MasR in brain tissue [24]. Another study revealed that not only does ischemic stroke result in increased levels of Ang (1–7), ACE2, and MasR, but the expression of AT1R was shown to be decreased in the ischemic cortex following transient MCAO in rats [21]. It is noteworthy that such a relationship between the ACE2-Ang (1–7)-MasR axis and stroke is also found in humans, in that serum levels of ACE2 were higher in human samples from cardioembolic stroke patients. Taken together, these studies suggest that alterations in the level of ACE2 might be a useful evidentiary and diagnostic marker in predicting stroke outcomes [21]. Furthermore, the beneficial outcomes of activation of the central ACE2-Ang (1–7)-MasR pathway in ischemic stroke could be explained both by increases in Ang 1–7 levels as well as reductions in Ang II levels [23].

The ACE2-Ang (1–7)-MasR axis may be exerting its protective effects against ischemic stroke through multiple mechanisms and may reflect diverse sites of action for Ang (1–7) [21,23,24,38,39,40,41]. Furthermore, such protection is not limited to stroke, but has also been demonstrated in a variety of inflammation-related cardiovascular diseases including hypertension, myocardial infarction, heart failure, cancer [21,26]. In this section, we will focus more on potential mechanisms of Ang (1–7)-induced neuroprotection in ischemic stroke.

Anti-inflammation and anti-oxidation responses. Several studies in the setting of stroke, have reported that activation of MasR by Ang (1–7) produces anti-inflammatory and anti-oxidative effects [21,22,41,42]. Regenhardt et al. showed that intracerebroventricular (ICV) administration of Ang (1–7) in an ischemic stroke model blunted the increased levels of pro-inflammatory cytokines, e.g., interleukin (IL)-1α and IL-6, and chemokines e.g., CXCL12 and its receptor CXCR4, in addition to inhibiting microglia/macrophage activation within the cerebral cortex [22,25]. The expression of the microglial marker CD11b was also attenuated by Ang (1–7) treatment in an ET1-induced ischemic stroke model [22]. Other pro-inflammatory factors, such as tumor necrosis factor (TNF)-α, IL-1β, and cyclooxygenase-2, were also reduced in the peri-infarct region following stroke by Ang (1–7) injection. These beneficial effects of Ang (1–7) were reversed by the MasR antagonist A-779, confirming that Ang (1–7) exerts its effects through binding and activating the MasR [22]. Moreover, Ang 1–7 has been reported to attenuate activation of the transcription factor and inflammation regulator NF-κB and its participation in TNF-α signaling. Inhibition of NF-κB translocation into the nucleus by Ang (1–7) resulted in decreased expression of proinflammatory cytokines, downregulated endothelial cell adhesion molecules, and reduced leukocyte chemotactic signaling, rolling, and adhesion [21]. These effects may particularly limit excessive inflammatory cell transmigration across the dysfunctional BBB. Alongside the potential direct neuroprotective actions of the ACE2-Ang (1–7)-MasR axis, this blunting of the vascular inflammatory response is likely to be critical for recovery from stroke.

Levels of iNOS, which can be a pro-oxidant enzyme, are decreased by the activation of the Ang (1–7) axis, and this subsequently decreases oxidative stress and limits neuronal cell death [21]. Ang (1–7) is also associated with attenuated oxidative stress in the ischemic brain through the elevated activity of superoxide dismutase, lower levels of malondialdehyde and decreased lipid peroxidation [22,23,25]. In addition to Ang (1–7) treatment, gene-engineered mice overexpressing ACE2 in neurons revealed a reduction in the levels of Nox2/Nox4 [43] and subsequent alleviation of ROS production after ischemic stroke [21] possibly through inhibition of microglial activation in cerebral cortex [21,25].

Vasodilation and angiogenesis. Findings from several studies indicate that the ACE2-Ang (1–7)-MasR axis exerts potent vasodilatory effects that maintain or increase local CBF in the post-ischemic brain by stimulating the production of eNOS and NO [21,23]. NO generated by eNOS exerts an acute protection against brain injury by improving CBF through increasing bradykinin secretion and promoting vasodilation in the penumbra area of the ischemic brain [23,25].

In addition to its vasodilator functions, ACE2-Ang (1–7)-MasR also confers vasoprotective by improving several other endothelial functions which are important in maintaining vascular homeostasis [21,44]. For example, an angiogenic role for the ACE2-Ang (1–7)-MasR pathway has also been described; ACE2 overexpression in endothelial progenitor cells enhances the angiogenic capacity within the penumbra region of the post-ischemic brain [21,44]. Intracerebroventricular (ICV) injection of Ang (1–7) also significantly increases brain capillary density via promoting endothelial cell proliferation, which is associated with activation of the MasR/eNOS pathway and upregulation of NO [21,40], while ACE2-overloaded neurons have been shown to stimulate the secretion of angiogenic cytokines (e.g., VEGF-A, FGF) in addition to improving CBF [21,40,43]. In summary, ACE2-Ang (1–7)-MasR-mediated increases in perfusion and angiogenesis could be of significant benefit in the setting of ischemic stroke.

Mas R anti-AT1R effects via altered kinase-phosphate signaling. MasR acts as a direct antagonist of AT1 receptors by making hetero-oligomers with AT1R, which is expressed in the neurons, microglia, and endothelial cells [21,33]. It has been reported that MasR physically alters the conformational constraints on AT1 signaling within the hetero-oligomer, leading to reduced receptor functionality [33]. Very recent studies specifically suggested that Ang (1–7) also reverses the deleterious effects of Ang II binding to AT1R via regulation of downstream kinase signaling [21,45]. Ang (1–7)-induced phosphatase activity mediates the beneficial effects of ACE2-Ang (1–7)-MasR by regulating cytokine and growth factor signaling (e.g., disruption of NF-κB pathway, as described earlier), and inhibiting AT1R-coupled signaling [21]. In general, activation of the AT1R by Ang II initiates mitogen-activated protein kinase (MAPK)-associated pathways, such as the AT1R-extracellular signal-regulated kinase (ERK)-p38 MAPK pathway, which result in upregulation of ACE and downregulation of ACE2 [21,46]. Conversely, it has been reported that administration of an ACE2 activator, Diminazene, significantly downregulates the phosphorylation of MAPKs such as ERK, p38, and c-Jun [21]. Furthermore, using VSMCs from rat thoracic aorta, Gallagher et al. reported that Ang (1–7) blunts Ang II-induced decreases in ACE2 expression via MasR activation [45]. This effect was found to depend on MAPK phosphatase activity, which inhibits MAP kinase [45,47]. Thus, we might conclude that the beneficial effects of Ang (1–7) reflect both phosphatase activation and subsequent downregulation of Ang II-induced MAPK signaling pathways (to prevent the harmful effects of Ang II-AT1R), as well as enhancement of the protective functions of the ACE2-Ang (1–7)-MasR axis.

Mas R interactions with bradykinin and AT2Rs. Accumulating evidence suggests that MasR may interact with several other receptors, especially the bradykinin and AT2R receptors [21]. The kinin–kallikrein system (KKS) produces bradykinin whose function is mediated by two bradykinin receptors, B_1_ (inducible) and B_2_ (constitutive) [37]. There is a complex interplay between the RAS and KKS, in which ACE cleaves and inactivates bradykinin [21,48]. Current studies indicate that the Ang (1–7)-MasR axis potentiates the vasodilatory function of bradykinin in cerebral ischemia through several mechanisms including increased expression of KKS components, facilitating its neuroprotective effects, and preventing its metabolism [21,37]. For example, central administration of Ang (1–7) in stroke models significantly increases levels of bradykinin, and upregulates bradykinin B_1_ and B_2_ receptors [37]. The vasorelaxant effects of both bradykinin and Ang (1–7) appear to be mediated by phosphorylation and activation of NOS [48].

A growing body of evidence supports the possibility of a connection between MasR and AT2R which could also contribute to the neuroprotection potential of Ang (1–7) in the setting of ischemic stroke [21]. AT2R-dependent functions of Ang (1–7) have been revealed by administering AT2R antagonists, such as PD123319 and PD123117, which result in the attenuation of Ang (1–7)-mediated prostaglandin synthesis, loss of coronary artery vasodilation, and increased mean arterial pressure [21,49]. Additional evidence for potential interaction, perhaps even heterodimerization of RAS and corresponding receptors, implicates interactions between AT1R and MasR, AT2R, and bradykinin B_2_ receptors [21,32]. Indeed, heterodimers of AT2R with bradykinin B2 receptor may also increase NO production [32]; while AT2R may antagonize AT1R via direct binding [50]. Similar to AT2R, MasR can serve as an AT1R antagonist by forming a heterodimer complex which can further attenuate the deleterious effects of Ang II [33].

Regarding potential interactions between MasR and AT2R, it is also worth considering how the anatomic localization and expression of MasR and AT2R might influence their direct interactions [21]. As mentioned earlier, MasR is expressed in microglia, neurons, and endothelial cells, while AT2R is primarily found within neurons and endothelial cells [21]. Following ischemic stroke, there is an elevated activation of microglial cells, as well as an increased expression of AT2R in the penumbra area of the infarcted tissue [51]. This upregulation could possibly result in increased MasR-AT2R interactions on neurons or microglia, which could intensify their protective functions [21].

Anti-vasoproliferative and hypertrophic actions. The activation of Ang II/AT1 signaling has been shown to be involved in vascular hypertrophy and remodeling by inducing VSMC growth, differentiation and proliferation [31]. Conversely, administration of human recombinant ACE2 (hrACE2) suppresses VSMC proliferation and vascular hypertrophy, and this is linked to inhibition of the JAK/STAT3 signaling pathway. Moreover, overexpression of ACE2 has been reported to reduce the expression of actin-binding protein profilin-1 which has been implicated in VSMC proliferation and vascular hypertrophy mediated by enhanced phosphorylation of Akt and ERK1/2/MAPK signaling cascades [31].

Anti-vascular fibrotic influence of ACE2/Ang 1–7. The ACE2/Ang 1–7 axis has also been reported to negatively modulate vascular fibrosis by controlling fibroblast density, fibrogenic pathways, and the production of extracellular matrix (ECM) proteins, such as collagens and matrix metalloproteinases (MMPs). Several studies have revealed that the anti-fibrotic role of ACE2 is mediated by down-regulation of MMPs, as increased activities of MMPs, particularly MMP-2 and MMP-9, contribute to the synthesis and deposition of ECM proteins. ACE2 also blocks the expression of fibrosis-associated genes, including transforming growth factor-β and connective tissue growth factor, which result in fibroblast proliferation, ECM deposition and cell migration, all known to be deleterious consequences of the Ang II/AT1 receptor pathway [31].

## 5. Pathology of Ischemic Stroke

**Figure 3:**
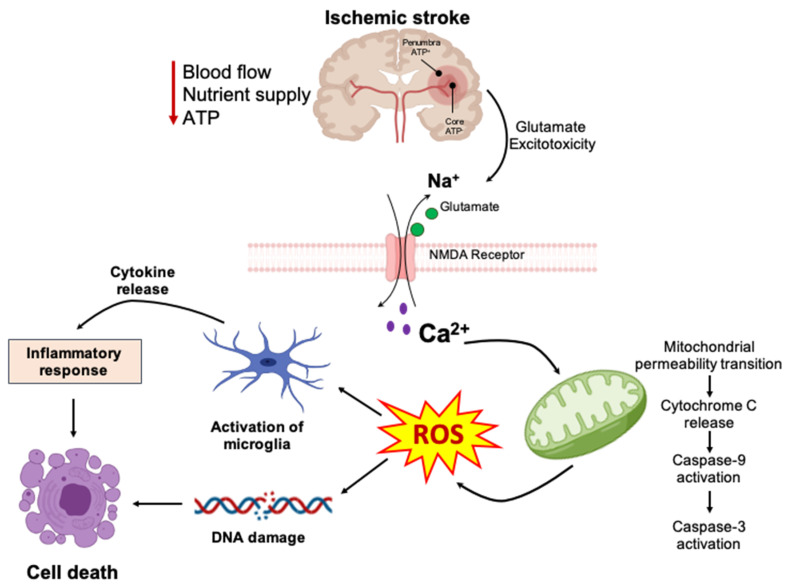
Pathophysiological mechanisms involved in ischemic stroke. The reduction in blood flow, nutrient supply, and energy as a result of cerebral artery occlusion leads to many complex pathophysiological events including energy failure, glutamate excitotoxicity, elevation of intracellular Ca^2+^ levels, peri-infarct depolarization (spreading depression), impairment of mitochondria function, generation of reactive oxygen species (ROS), activation of microglia, secretion of pro-inflammatory cytokines/chemokines, inflammatory responses, and cell death.

The ischemic cascade is rapidly initiated within seconds to minutes after severe focal hypoperfusion [17] (Figure 3). The reduction in CBF as a result of cerebral artery occlusion by an embolus or by local thrombosis leads to many complex pathophysiological events including energy failure, excitotoxicity, elevation of intracellular Ca^2+^ level, peri-infarct depolarization (spreading depression), generation of ROS, BBB disruption, post-ischemic inflammatory responses and cell death [8,52,53]. While reperfusion of the ischemic region is critical for restoring normal neuronal function, it can paradoxically result in secondary damage known as ischemia reperfusion injury [17]. All of these events exacerbate the initial injury, which will further lead to permanent cerebral damage [17].

### 5.1. Blood Flow in the Post-Stroke Brain

Because the brain lacks oxygen and nutrient reserves, adequate CBF is crucial for maintaining a continuous supply of oxygen and nutrients to this organ [54]. There are multiple overlapping regulatory mechanisms required to maintain CBF including cerebral autoregulation, flow-metabolism coupling (functional hyperemia), and neurogenic regulation, involving multiple cell types (endothelial and VSMCs, neurons, and astrocytes) [55,56].

Cerebral autoregulation is the primary physiological mechanism that preserves relatively constant CBF within the physiologic range over a wide range of blood pressures or intracranial pressures [55,57,58] and it involves coordinate myogenic, neurogenic, and metabolic regulatory mechanisms [56,57]. When mean arterial pressure (MAP) drops below 50 mmHg, cerebrovascular resistance is decreased, invoking maximum vasodilation to enhance perfusion. In contrast, when MAP exceeds 150 mmHg, intense arteriolar vasoconstriction minimizes elevations in hydrostatic pressure that might cause cerebral edema and disruption of BBB [58].

Superimposed on autoregulation is ‘functional hyperemia’, which matches the delivery of blood flow to the activity level of each brain area [54]. Several vasodilating molecules (K^+^, H^+^, adenosine, and NO) are increased by elevated synaptic transmission as well by increased arterial pCO_2_ and, to a lesser extent, decreased arterial pO_2_, and play critical roles in coupling CBF with metabolism [54,55]. Neurogenic regulation is mediated by perivascular nerves, which innervate vessels outside of the brain parenchyma extrinsically, or connect to astrocyte end feet intrinsically [55].

Endothelial cells are dynamic sources of NO, EDHF, eicosanoids, and ET-1, which influence CBF regulation. Endothelial cells can promote vasodilation or constriction in response to shear stress and transmural pressure through integrin–matrix–mechanoreceptor modulated events [55]. Astrocyte end feet also physically link cerebral microvasculature with synapses to modulate neuronal and vascular function. Astrocytes induce vasodilation predominantly through K^+^ and cyclooxygenase-1 reaction products [54,55].

Unlike extra-CNS microvascular beds, during acute phases of stroke, ischemia negatively impacts endothelial and smooth muscle cells, leading to impaired cerebral autoregulation [57,59]. Such changes can at least temporarily convert the normal, relatively flat portion of the brain autoregulatory curve to a more linear relationship. Changes in BP under conditions of impaired cerebral autoregulation might therefore result in hemorrhagic transformation, brain edema, and further ischemic injury [57,58]. For instance, the ischemic vessels that have been dilated in response to reduced blood flow (hypoperfusion), lose their capability to quickly adjust with increased blood flow (reperfusion) following recanalization [57]. Bleeding induced by the loss of autoregulatory constriction can create serious problems over time through iron deposition; if bleeding is significant, in the sealed compartment of the skull, hemorrhagic transformation can increase pressure, with more widespread and disastrous effects on regional perfusion.

### 5.2. Progressive Post-Stroke Vasoconstriction

There appears to be a conversion from loss of autoregulation to intense vasoconstriction in the ischemia-affected region, which may lead to the sacrifice of hemorrhage-prone brain regions in an effort to ultimately preserve more long-term global perfusion. It is likely that even when thrombi/emboli are successfully removed by endarterectomy or dissolved by tPA, there is still an intense and remnant vasoconstriction, which gradually intensifies within the first 24 h of the ischemic stress [20,60]. It is however striking that within the first 4 h following relief of the ischemic occlusion in the MCAO model, brain structure and function is still essentially preserved [60]. The typical loss of brain tissue, observed as infarcted tissue at 24 h reperfusion, therefore occurs progressively *after* the first 4 h and not before. This makes sense in the context of conventional tPA administration guidelines, where treatment is effective only if given during the first few hours of stroke. Although the exact mechanisms through which post-stroke vasoconstriction occurs requires further work, Hall et al. have provided a provocative hypothesis about the role for pericytes [61]. Consistent with our findings, they showed that during ischemia, there is an initiation of pericyte constriction followed by death of these important vasoregulatory cells [62,63]

### 5.3. Pericytes Constrict Capillaries and then Die in Rigor

The relationship between increased CBF and brain activity involves coordination of several levels of vascular dynamics in response to demands of neuronal processing. Most nerve cells are in close proximity to capillaries [64], with intervening pericytes that receive signals from both vascular endothelial and parenchymal neurons, as well as astrocytes and glia. In response to factors, such as norepinephrine, glutamate, NO, hydrogen sulfide, and eicosanoids, pericytes and VSMCs influence blood flow at the level of precapillary arterioles and capillaries.

Vasodilation/vasoconstriction responses can spread between apposed pericytes [62,65] and possibly between pericytes and arteriolar (and venular) smooth muscle in response to autacoids and their downstream second messengers. Hall et al.’s study using BOLD functional imaging signals demonstrated that capillary dilation is controlled by pericytes [61]. They reported that, at sites of pericyte coverage, capillaries dilate faster and more strongly than arterioles. This suggests that pericytes relax tonic constriction to increase capillary perfusion. Hall et al. further proposed a model where, when compared to arterioles, perivascular/parenchymal pericytes react more rapidly and intensely to increased neuronal activity, and that pericytes can then convey a secondary hyperpolarizing vasodilatory impulse to upstream arterioles via pericyte–endothelial connexins [62,65]. However, direct capillary vasodilation provides a larger degree of regional microvascular blood flow control, with capillary dilation estimated to generate 84% of the steady-state increase in blood flow evoked by neuronal activity.

It was previously envisaged that tonic capillary constriction in otherwise healthy pericytes could be reversed by relieving oxidative stress [63]. However, this does not seem to occur in ischemic stroke. Hall et al. found that, after ischemia there is an initiation of permanent pericyte constriction and death [61], known as ‘death in rigor’, i.e., these constricted pericytes do not recover. These pericyte changes represent key events opposing the restoration of CBF, even when arteries are unblocked after stroke by interventions e.g., TPA or endarterectomy [66,67]. While the reasons for pericyte death are not yet fully understood, it appeared to be mediated in part by glutamate. In contrast, antioxidants are not effective, possibly reflecting independent underlying mechanisms controlling constriction versus death.

Not only will ‘pericyte death in rigor’ provoke persistent or permanent loss of capillary blood flow [66,67], but it will also eliminate pericyte-dependent blood–brain barrier mechanisms [68,69]. The loss of these normal pericyte controlled functions can be predicted to contribute to progressive neuronal damage following stroke. It is noteworthy that we found this progressive loss of perfusion is prevented by PMSCs through an ACE2-dependent pathway [20]. Our findings along with those of Hall et al. invoke the possibility that accumulation of Ang II and/or insufficient Ang 1–7 could contribute to loss of pericyte survival, and that this cascade of events is at least in part responsible for the impaired CBF that continues following reperfusion in a stroke patient. This is further supported by previous work showing that Ang II, via ATR1, stimulates glutamate release [70], whereas an ACE2 activator can reduce the excitatory synaptic activity of neurons, in association with decreased expression of the NMDA receptor (ionotropic glutamate receptor) [71]. Thus, it is plausible that the shift in balance between the ACE-Ang II-AT1R and ACE2-Ang (1–7)-MasR axes during stroke leads to increased glutamate signaling that Hall et al. showed contributed to pericyte death.

## 6. COVID-19 Related Ischemic Stroke

With respect to COVID-19 pathophysiology, because the SARS-CoV-2 virus infects endothelial cells using ACE2 as a binding receptor and triggers internalization of ACE2, the normal catabolism of Ang II to Ang1–7/Ang1–9 in the vascular compartment is inhibited during infection with this virus [72,73]. Recent studies have suggested that endothelial ACE2 deficiency activates and intensifies hyper-coagulation pathways due to the dysregulation between the ACE-Ang II-AT1R and ACE2-Ang 1–7/1–9-MasR/AT2R axes [74,75,76,77]. In agreement with this, Verdecchia et al. showed that ACE2 catalytic capacity is lost upon SARS-CoV-2 penetration of endothelial cells [77], leading to derangement of several endothelium-mediated functions, including vasoregulation, anti-inflammation, and anti-thrombosis responses. Such loss of ACE2 activity may increase stroke risk and provoke the other thrombotic complications seen in COVID-19.

Interestingly, COVID-19 patients usually present with elevated platelet numbers, highly increased fibrinogen and slightly prolonged prothrombin and activated partial thromboplastin time [78,79]. These observations clearly suggest that in the brain, SARS-CoV-2 infection of endothelial cells may result in cerebral endothelial dysfunction, inflammation and heightened pro-coagulant state, culminating in the intensified microvascular stroke pathology often seen in COVID-19 patients [72,80]. Early studies indicate that this holds true, with ischemic and hemorrhagic stroke being complications in at least 6% of COVID-19 patients. Moreover, in these patients, endothelial activation has been shown to be associated with elevated circulating markers of coagulation (e.g., D-dimers) [81,82,83,84] and inflammation (e.g., TNF-α, IL-6, IL-2, and monocyte chemoattractant protein-1) [85]. While there is a growing movement to treat COVID-19 patients with anticoagulants prophylactically to prevent thrombotic events, this approach does not hold for primary stroke prevention, and comes with increased bleeding risk [86]. This raises the question of whether COVID-19-associated stroke differs from stroke in non-COVID-19 patients. However, the current data in COVID-19 patients is preliminary and correcting the underlying mechanisms leading to intensified thrombosis may be a more effective alternative, particular for seriously ill COVID-19 patients.

While anti-thrombotic and anti-inflammatory properties of hPMSCs-derived ACE2 in the setting of brain vascular endothelium or SARS-CoV-2 infection have not yet been tested, this previously understudied effect of ACE2, and ACE2 products, remains an extremely important axis that may be exploited therapeutically, for example, to treat COVID-19 patients using hPMSCs that express, and are a source of, ACE2. Because COVID19-mediated stroke, and experimental stroke models are both thrombotic phenomena exhibiting deficits in ACE2/MasR signaling, the successful demonstration that SARS-CoV-2-induced ACE2 suppression mediates increased thrombotic risk and that stem cell ACE2/MasR activators may eliminate such risk could define highly novel, valuable, innovative, and safe approaches for clinically managing COVID-19 stroke, and other thrombotic complications of COVID-19 (pulmonary embolism).

## 7. Discussion

Our previous findings showed that CBF recovery after stroke is improved by PMSCs, and that this protection is mediated through the ACE2-Ang (1–7)-MasR pathway. While this was initially somewhat surprising, our data are consistent with many existing paradigms showing the vasoregulatory balance between ACE and ACE2 pathways. The literature thus suggests that interventions that might limit the progressive vasoconstriction in ischemic stroke could have enormous benefits on post-stroke outcomes. Because we have seen that this progressive vasoconstriction: (1) occurs later than 4 h; (2) is relieved by ACE2 mediated mechanisms; and (3) is MasR dependent, we have now identified ACE2 as a powerful and novel approach to prevent stroke progression mediated by post-occlusive vasoconstriction. Moreover, studies by Hall et al. have emphasized the enormous importance of controlling pericyte death as a possible therapeutic modality following stroke. Although not yet directly correlated with pericyte death, our recent findings in stem cells-ACE2 dependent stroke therapy remain wholly consistent with Hall et al.’s concept that ‘death in rigor’ is in fact the same as our progressive vasoconstriction in stroke, it is still unclear whether, and to what extent, Ang II contributes to this injury phenomenon. Similarly, it is also unclear to what extent ACE2 metabolites, e.g., Ang 1–7/Ang 1–9 rescue of this phenomenon reflects prophylactic stabilization of pericytes against ‘death in rigor’.

It is worth noting that, in addition to improved CBF, enhancement of the ACE2-Ang 1–7/1–9-MasR/AT2R pathway likely also protects against post-stroke injury through other pathways, including decreased inflammation and further thrombotic complications and, thus, represents a potential therapy with pleiotropic effects. However, it is the critical step of protecting CBF that may confer the most promise for therapies designed to stimulate the ACE2-Ang 1–7/1–9-MasR/AT2R pathway, because of the fact that, after thrombolysis is effectively achieved using tPA and, therefore, significant brain stress has already been induced, there remains disastrous and progressive vasoconstriction. Therefore, forms of therapy beyond simple “reperfusion” are likely required to ‘prop’ open these vessels until the brain is able to rebalance perfusion and normal vascular control. Furthermore, careful design of the therapy is crucial, as it is apparently not sufficient to simply suppress formation or action of Ang II via AT1R blockade or ACE inhibitor, because they also block formation of the beneficial species formed by ACE2. We have evidence that administration of PMSCs, or exosomes derived from PMSCs, to a stroke model enhances the ACE2-Ang 1–7/1–9-MasR/AT2R pathway, while circumventing issues of injecting immunogenic stem cells, inadvertent blockade of the ACE2 cascade, and failure of treatments that target pathways leading to stroke injury because CBF has not been adequately restored. Of particular interest during the COVID-19 pandemic is the possibility that loss of pericyte ACE2 via pericyte stress/death would lead to wider and more extensive vasoconstriction, and therefore ACE2 dynamics could be important not only in the stetting of stroke but also in COVID-19 pathophysiology. Enduring neurological disturbances in ‘long-COVID’ might reflect Ang II/ACE2 metabolism, which influences pericyte control of CBF, as well as inflammatory and thrombotic cascades. However, clinical studies, and ultimately trials, will need to be performed to confirm our hypothesis, and test the feasibility of targeting the ACE2/Ang (1–7)/MasR axis in human stroke patients.

## 8. Conclusions

In conclusion, while many studies focus on injury mechanisms producing the infarct, we may now be able to, for the first time, prevent stroke progression from happening in the first place. It has been reestablished that use of ACE2 pathway activators in conjunction with the tissue plasminogen activator, as soon as possible after reperfusion, can significantly improve outcomes, which would otherwise become irreversible. The added benefit of blocking other responses, such as inflammation, can only serve to provide an even better recovery following stroke.
